# Confinement induces helical organization of chromosome-like polymers

**DOI:** 10.1038/s41598-018-37261-8

**Published:** 2019-01-29

**Authors:** Youngkyun Jung, Bae-Yeun Ha

**Affiliations:** 1 Supercomputing Center, Korea Institute of Science and Technology Information, Daejeon, 34141 Korea; 2 Department of Physics and Astronomy, University of Waterloo, Waterloo, Ontario N2L 3G1 Canada

## Abstract

Helical organization is commonly observed for a variety of biopolymers. Here we study the helical organization of two types of biopolymers, i.e., DNA-like semiflexible and bottle-brush polymers, in a cell-like confined space. A bottle-brush polymer consists of a backbone and side chains emanating from the backbone, resembling a supercoiled bacterial chromosome. Using computer simulations, we calculate ‘writhe’ distributions of confined biopolymers for a wide range of parameters. Our effort clarifies the conditions under which biopolymers are helically organized. While helical organization is not easily realized for DNA-like biomolecules, cylindrical confinement can induce spiral patterns in a bottle brush, similarly to what was observed with bacterial chromosomes. They also suggest that ring-shape bottle brushes have a stronger tendency for helical organization. We discuss how our results can be used to interpret chromosome experiments. For instance, they suggest that experimental resolution has unexpected consequences on writhe measurements (e.g., narrowing of the writhe distribution and kinetic separation of opposite helical states).

## Introduction

Biopolymers show a variety of conformational behavior, driven by intermolecular interactions, external perturbations, or physical constraints. Helical organization of biopolymers is relevant in a number of contexts. For instance, alpha helices are commonly observed for proteins^1^. Collagen molecules are helically organized (see ref.^2^ and relevant references therein). Spiral patterns are a key feature of bacterial chromosomes at large scales^3–6^. A related phenomenon is the helical organization of hard spheres in a cylindrical space, induced by self-crowding^7^.

Of particular interest is spontaneous helical organization of biopolymers induced by cylindrical confinement. Indeed, bacterial chromosomes accomplish the formidable task of organizing themselves into a well-ordered structure in a highly confined cellular space^8–10^. ‘Length-wise’ folding characterizes chromosome organization in elongated bacteria^8–10^. It can benefit from the helical organization of the chromosome along the long axis of the cell. This is in part accomplished by the action of various chromosome-associated proteins. How their “local” action influences the large scale organization of the chromosome, as desired for their function, is not obvious^10^. A better understanding of large-scale chromosome organization will necessitate a systematic study of model chromosomes: coarse-grained chromosomes and their chromosome-like organization in a confined space^9–12^.

**Figure 1:**
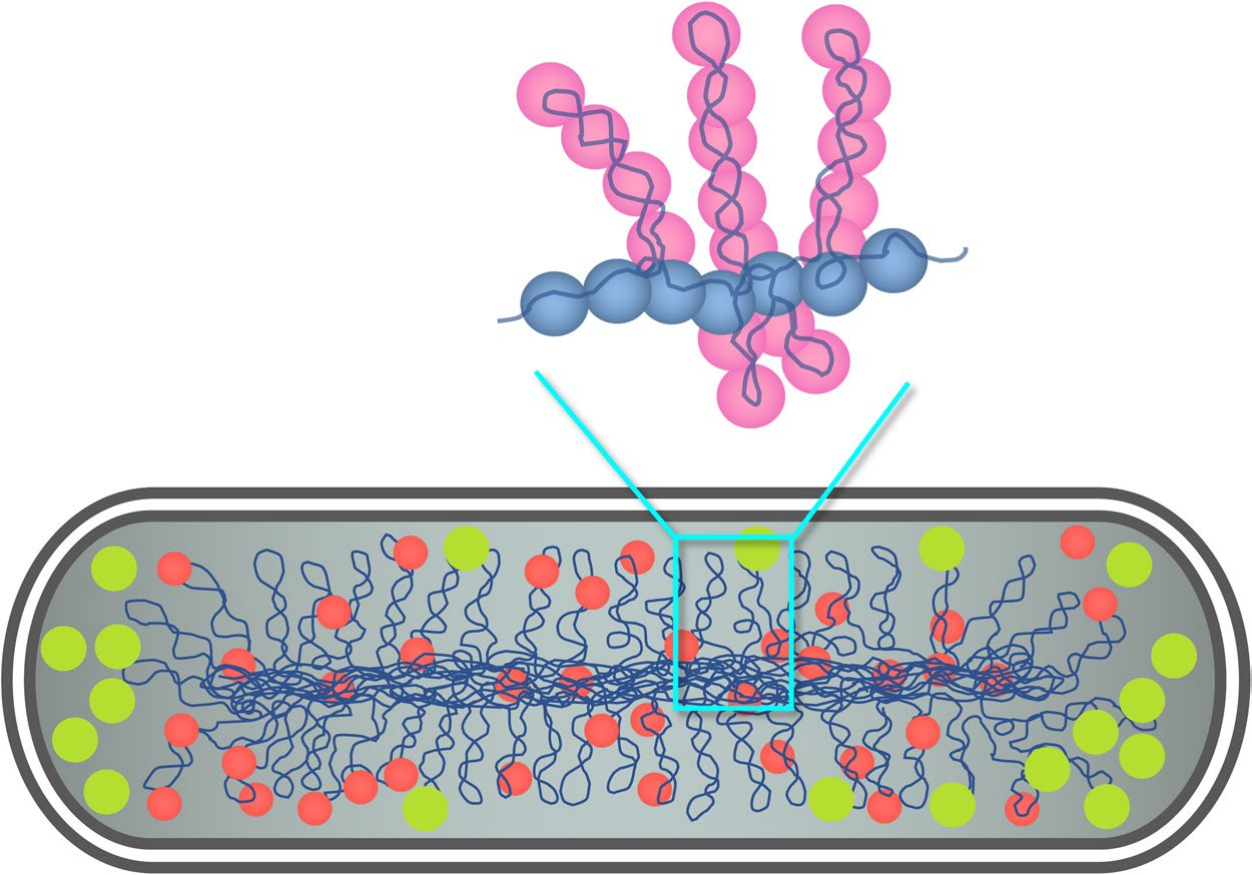
Schematics of a bacterial chromosome (in dark blue) and a bottle-brush polymer (top panel). Chromosome-associated proteins are shown in red and ribosomes in light green. For simplicity, topological and conformational complexities arising from DNA replication and transcription are ignored. Also because of phase separation between the chromosome and crowders (e.g., ribosomes), the chromosome can be viewed as being confined in a sub-cellular space with a reduced volume. The bottle-brush polymer consists of a backbone chain formed by monomers in dark blue and side loops (supercoiled plectonemes) spreading out from the backbone. The bottle brush was employed as a model Caulobacter chromosome^3^. This illustration is in part based on refs^3,34,35^.

Using molecular dynamics simulations, we study the helical organization of model biopolymers: DNA-like semiflexible chains and bottle-brush polymers confined in a cell-mimicking cylindrical space. A bottle-brush polymer consists of a backbone and side chains or loops spreading from it. It resembles supercoiled bacterial chromosomes as schematically shown in Fig. 1. In the figure, some details such as topological and conformational complexities arising from DNA replication and transcription are ignored. Also the chromosome and crowders (e.g., ribosomes) are well segregated. The chromosome can then be viewed as being confined in a cylindrical sub-cellular space with a reduced length and diameter. Indeed, a bottle-brush polymer, employed as a coarse-grained model of bacterial chromosomes^3^, was shown to be consistent with into Hi-C data^3^ (see refs^13,14^ for bottle brushes in different contexts).

Our main focus is on clarifying how confinement induces helical organization in an otherwise non-helically organized biopolymers. Some biopolymers, especially double-stranded DNA, have intrinsic propensity to be helically organized along its backbone; in DNA, two adjacent base pairs are twisted against each other into a helix^15^. In our coarse-grained modelling, this kind of feature is not taken into account. Instead, we present a physical picture of helical organization at large scales observed with bacterial chromosomes (see Fig. 1). The main goal is to make a quantitative sense of this observation^3–6^. To this end, we calculate ‘writhe’ (Wr) and Wr distributions of model biopolymers for a wide range of parameters^16^. Conceptually, writhe measures the number of times a one-dimensional object (e.g., a polymer chain or a curve) ‘crosses over’ itself^15^.

Our results clarify the conditions under which biopolymers are helically organized. Helical organization is not easily realized for DNA-like semiflexible polymers (beyond their intrinsic tendency to be double helices). For instance, for typical parameters for DNA, their helical organization requires unrealistically large persistence lengths. Beyond double helical structure, DNA-like molecules by themselves will not be helically organized by confinement. This finding needs to be contrasted with the recent work, which shows how DNA-like molecules are helically organized^17^. In contrast, cylindrical confinement can easily induce spiral patterns in a bottle brush polymer which would otherwise remain randomly organized. This is an entropically driven phenomenon: the side chains tend to be aligned parallel with each other in helical patterns so as to maximize their conformational entropy^18,19^. Our results also suggest that ring-shape bottle brushes have a stronger tendency for helical organization, compared to the corresponding linear bottle brushes.

A distinguishing feature of typical confined bottle brushes is that left-handed and right-handed helical states are kinetically well-separated from each other. A bottle brush tends to get trapped either in a negative or positive helical state, even if both are equally likely populated in equilibrium. Caution thus needs to be used in interpreting Wr measurements, since this feature will be washed out in a population average of Wr, i.e., an average over all states. Instead, one can sample in a sub-ensemble presented by either negative or positive helical states.

Finally, we discuss how our results can be used to interpret better chromosome experiments^3–6^. For instance, they indicate how limited resolution can alter Wr measurements; the lower the resolution is, the narrower the Wr distribution is. Consequently, limited resolution or coarse-graining will enhance the kinetic separation of opposite helical states. Based on our results, we interpret the propensity of helical organization seen in a confined bottle-brush polymer as benefiting the lengthwise folding of bacterial chromosomes^8^.

## Simulation Procedure

In our molecular dynamics (MD) simulations, we use the bead-spring model of a polymer chain: beads or monomers of size *σ* each. Let *r* be the center-to-center distance between beads. Adjacent beads are connected to each other through the finitely extensible non-linear elastic (FENE) potential:

1 $${U}_{{\rm{FENE}}}(r)=-\,\frac{1}{2}k{r}_{0}^{2}\,\mathrm{ln}[1-{(\frac{r}{{r}_{0}})}^{2}],$$

where the spring constant *k* and the range of *U*_FENE_(*r*) are set to *k* = 30.0*ε*/*σ* and *r*_0_ = 1.5*σ*^20,21^. The beads interact with each other through the fully-repulsive Weeks-Chandler-Anderson (WCA) potential, which is given by

2 $${U}_{{\rm{WCA}}}(r)=\{\begin{array}{ll}4\varepsilon [{(\frac{\sigma }{r})}^{12}-{(\frac{\sigma }{r})}^{6}+\frac{1}{4}] & {\rm{for}}\,r < {2}^{\mathrm{1/6}}\sigma \\ 0 & {\rm{otherwise}}\end{array},$$

where *ε* and *σ* represent the strength and range of the WCA potential, respectively^22^. The parameter *σ* can be designated as the size of each bead or monomer. In the polymer physics community, ‘*a*’ is traditionally used for the bead or monomer size^23^. Here we use *σ* and *a* interchangeably, preferentially *σ* when referring to the simulation procedure and *a* otherwise.

The chain stiffness is taken into account by a harmonic bending potential

3 $${U}_{{\rm{bend}}}={k}_{b}{(\theta -\pi )}^{2},$$

where *θ* is the angle between two successive bond vectors of a chain^24^. A prefactor *k*_*b*_ is related to the persistence length as ${\ell }_{{\rm{p}}}\mathrm{=2}{k}_{b}\langle b\rangle /{k}_{{\rm{B}}}T$ with the mean bond length $\langle b\rangle \simeq 0.97\sigma$ and the thermal energy *k*_B_*T*. Here, *k*_B_ is the Boltzmann constant and *T* the temperature.

The polymer chain is trapped inside a cylindrical space. The confining cylinder is made up of “imaginary” beads of size *σ*, interacting with beads through WCA potential given in Eq. 2.

The equation of motion for beads is integrated using the velocity-Verlet algorithm with a time step 0.005*τ*, where $\tau =\sigma \sqrt{m/\varepsilon }$ (*m* is the bead mass), while the system is kept at constant temperature *T* = 1.0*ε/k*_B_ via a Langevin thermostat with a damping constant *γ* = 1.0*τ*^−1^ ^21^. For our simulations, we used the simulation package LAMMPS (‘Large-scale Atomic/Molecular Massively Parallel Simulator’)^25^.

The semiflexible polymer was initially confined in a cylinder closed with pistons and compressed gradually until the piston-piston distance reaches a designated cylinder length. After equilibration for about 10^8^ time steps, we performed a simulation run for 2 × 10^8^ time steps and obtained data every 10^4^ time steps; the chain relaxation time obtained from the autocorrelation function of Wr was found to be ≈10^6^~10^7^ time steps depending on the chain stiffness.

Similarly, for the bottle-brush polymer, initially, the backbone monomers were arranged in either left-handed or right-handed helical state in a cylinder with the sidechain monomers collapsed onto each corresponding backbone monomer. The monomers were pushed apart from each other using a pair soft potential, *U*_soft_ = *A*[1 + cos(*πr*/*r*_*c*_)], where *r* is the distance between monomers, *A* is the interaction strength, and *r*_*c*_ is the cutoff length. After this procedure, the soft potential was turned off and the WCA potential was turned on. The bottle brush was then capped by pistons and gradually compressed with the pistons at a constant velocity (about 10^−3^*σ*/*τ*) to the desired cylinder length.

Unlike the semiflexible case, the motion of a bottle-brush polymer in a cylinder is hindered by its sidechains–more so for longer sidechains. Indeed, we note that the transition between opposite helical states is a rare event, even though they are equally probable in equilibrium. Sampling the entire right-handed and left-handed space is prohibitively long for a confined bottle brush with long sidechains, e.g., each consisting of 40 monomers. To circumvent this difficulty, here we employ a kinetically meaningful, “local-equilibrium” approach to the computation of writhe. We let the bottle brush equilibrate in either helical state: If initially in a left-handed helical state, the bottle brush will equilibrate in a subspace spanned by left-handed conformations. To this end, we let the system evolve for 2 × 10^8^ time steps, which turned out to be longer than the relaxation time of the backbone writhe in either subspace. After this, we ran additional 2 × 10^8^ integration steps, obtained a data point every 10^4^ steps, and averaged it over 20 independent simulations; in each simulation, a different random seed was used and thus the polymer evolved differently from the initial conformation.

This local equilibrium approach might be relevant for the writhe measurement of bacterial chromosomes. These highly confined biomolecules will experience similar kinetic effects. See the Results section for related discussions.

## Results

The helical organization of a biopolymer is often characterized by writhe (Wr). Writhe measures the extent of helicity of a (closed) curve *C* in space by counting the “signed” number of self-crossings made by the curve projected onto a plane^26^. Mathematically, the writhe of *C* can be given as the Gauss double integral along *C*. If **r**_1_ and **r**_2_ are the positions of loci on the curve and **r**_12_ = **r**_2_ − **r**_1_, Wr is given by

4 $${\rm{Wr}}=\frac{1}{4\pi }{\int }_{C}{\int }_{C}\frac{(d{{\bf{r}}}_{2}\times d{{\bf{r}}}_{1})\cdot {{\bf{r}}}_{12}}{{r}_{12}^{3}}\mathrm{.}$$

**Figure 2:**
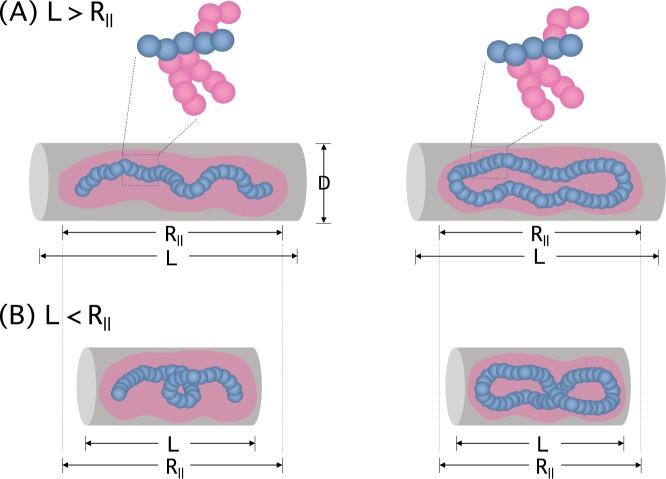
Bottle brush polymers in a cylindrical space of length *L* and diameter *D*: linear (left) and ring (right). In an open or long cylinder, the bottle brush stretches along the long axis of the cylinder as shown in (**A**), where the equilibrium length *R*_||_ of the confined bottle brush is larger than its size in free space. If *L* < *R*_||_, the bottle brush is compressed longitudinally and can be helically organized as evidenced below. According to the sign convention, the writhe of the backbone in (**B**) has a positive (left) and negative sign (right).

In a numerically-oriented approach, one can view the curve as a linear succession of many straight segments. Writhe can then be expressed as the double sum of the signed solid angle formed by two arbitrary segments, averaged over all possible segment directions^27^; here, the solid angle is the area of a parallelogram spanned by the two segments divided by the square of the distance between the beginnings of the two segments and the sign is set by the sign convention for the crossing between the segments, i.e., either left- or right-handed (see Fig. 2).

Let *L* and *D* be the length and diameter of a confined cylindrical space, respectively, as shown in Fig. 2. In our consideration, *D* is assumed to be smaller than the size of polymers when they are in free space. In this case, (open) cylindrical confinement tends to elongate the polymer (see Fig. 2(A) for a bottle-brush polymer)^28–30^. Let *R*_||_ be the equilibrium polymer length in the longitudinal direction when *L* = ∞. If *L* < *R*_||_, the polymer is compressed longitudinally (see Fig. 2(B))^29,31^. As evidenced below, this longitudinal compression is one of the determining factors for helical organization.

**Figure 3:**
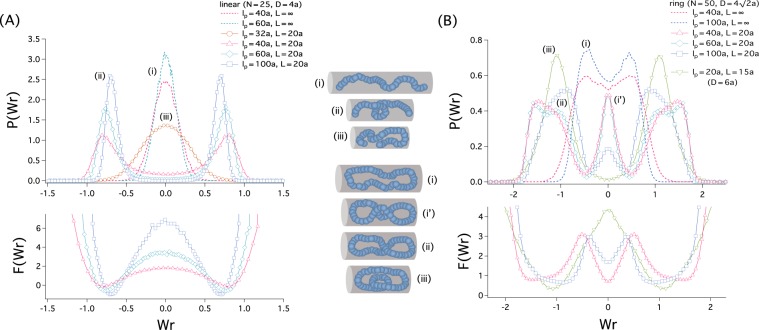
Confinement and helical organization of a semiflexible polymer with linear (**A**) and ring topology (**B**). The upper graphs show the probability distribution of Wr, *P*(Wr); the lowers ones display the corresponding writhe free energy, *F*(Wr) = −*k*_B_*T* ln *P*(Wr), given in units of *k*_B_*T*. Typical conformations, labelled as (i), .., and (iii), in the middle panel, represent various curves in the graphs; the corresponding Wr value is marked by (i), …, or (iii) in the graphs (e.g., Wr ≈ −1 for (ii) and Wr ≈ 0 for (iii)). (**A**) For the linear-chain case, we have chosen the cylinder diameter *D* = 4*a*, the number of monomers *N* = 25, and used several combinations of ${\ell }_{{\rm{p}}}$ and *L*. In all cases, the confined chain does not show preferred handedness (either left or right) in the sense that *P*(Wr) is symmetric about the *y* axis; as a result, 〈Wr〉 = 0, where 〈Wr〉 is a quantity averaged with respect to *P*(Wr), as is most obvious for the open-cylinder case or for *L* > *R*_||_ (see the illustration labelled as (i)). A distinguishing feature of the compressed case (*L* < *R*_||_) is the emergence of two-peaks in *P*(Wr) except for ${\ell }_{{\rm{p}}}=32a$. For a larger ${\ell }_{{\rm{p}}}$ value, the width of *P*(Wr) is smaller but the peak is slightly closer to the origin. The writhe free energy (lower panel) suggests that the barrier between negative and positive helical organizations is appreciable for a larger value of ${\ell }_{{\rm{p}}}$. Combined with the upper graph, this suggests that both stiffness and the degree of closed confinement are the key parameters for the helical organization of semiflexible polymers. (**B**) Similarly to the linear case, a ring polymer is helically organized under the right conditions: large ${\ell }_{{\rm{p}}}$ and small *L*/*R*_||_. We have chosen $D=4\sqrt{2}a$ and *N* = 50. The main difference between the linear and ring cases is two-fold: first, *P*(Wr) is double-peaked even for *L* > *R*_||_; also, *P*(Wr) develops a third peak at the origin for large ${\ell }_{{\rm{p}}}$ and small *L* values. The barrier height between left and right handedness is $\lesssim 2{k}_{\text{B}}T$. An emerging picture from this consideration is that while a stiff ring polymer tends to be helically organized, it can change its handedness relatively easily; for ${\ell }_{{\rm{p}}}=40a$, the middle peak is equally likely populated. On the other hand, when compressed sufficiently, the ring polymer is also back-folded, as described by the ring illustration (iii) in the middle column, corresponding to the green curve for *D* = 6*a* in the upper graph in (**B**).

Figure 3 displays our results for the probability distribution of Wr, denoted as *P*(Wr), and the free energy of Wr, given by *F*(Wr) = −*k*_B_*T* ln *P*(Wr), for a linear (A) and ring polymer (B); the free energy in the lower graph is given in units of *k*_B_*T*.

For the linear-chain case, we have chosen the cylinder diameter *D* = 4*σ* = 4*a*, the number of monomers *N* = 25, and used several combinations of ${\ell }_{{\rm{p}}}$ and *L*, as indicated in Fig. 3(A): ${\ell }_{{\rm{p}}}=32a,\,\mathrm{...100}a$ and *L* = 20*a*, ∞. Note that compression of a confined semiflexible chain leads to the formation of helices if $D\ll {\ell }_{{\rm{p}}}$^17^, as reflected in our parameter choices. Depending on the parameter choices, the graph of *P*(Wr) is either single- or double-peaked. The physical pictures represented by the curves on the graph are depicted in the middle panel: (i) non-helical, (ii) helical, and (iii) back-folded or hairpin-turn-like. The Wr value corresponding to each conformation is marked by (i),… or (iii) in the graph [e.g., Wr ≈ −1 for (ii) and Wr ≈ 0 for (iii)]. The helical organization in (ii) is characterized by the emergence of two peaks in *P*(Wr). It is clear that both closed cylindrical confinement and chain stiffness are key factors for helical organization. As the persistence length ${\ell }_{{\rm{p}}}$ increases from ${\ell }_{{\rm{p}}}\mathrm{=32}a$ to 100*a* for a fixed *L* = 20*a*, *P*(Wr) evolves from single-peaked to double-peaked distributions. In an open cylinder, i.e., *L* = ∞, the confined polymer is non-helically organized.

In all cases shown in the figure, *P*(Wr) is symmetric about the *y* axis; as a result, 〈Wr〉 = 0, where 〈Wr〉 is a quantity averaged with respect to *P*(Wr), as is most obvious for the open-cylinder case or for *L* > *R*_*||*_, as illustrated in the middle column (i). In the double-peak case, the two peaks are equally likely to be populated.

The writhe free energy given in units of *k*_B_*T* (lower panel in Fig. 3(A)) suggests that the barrier between negative and positive helical organizations is appreciable for a large value of ${\ell }_{{\rm{p}}}$. The confined polymer will get trapped in a local free energy minimum, either at a negative or positive Wr value; it is helically organized as described by the illustration (ii). On the other hand, for ${\ell }_{{\rm{p}}}\mathrm{=32}a$, the chain is back-folded because of a lower bending energy cost (see (iii)). This means that the helical organization of semiflexible polymers is favored by their bending energy, consistent with earlier studies^17^.

Figure 3(B) summarizes the simulation results for a ring semiflexible polymer. We have chosen $D\mathrm{=4}\sqrt{2}a$ (*D* = 6*a* as well) and *N* = 50, and used various choices of ${\ell }_{{\rm{p}}}$ and *L*. (When the ring polymer is viewed as a parallel connection of two ‘subchains’ or ‘arms,’ the choice of *D* here is motivated by the fact that with this choice each subchain occupies about the same cross-sectional area as in the linear case^32^.) Similarly to the linear case, a ring polymer is helically organized under the right conditions: large ${\ell }_{{\rm{p}}}$ and moderately-small *L*/*R*_||_ (ii). The main difference between the linear and ring cases is two-fold. First, *P*(Wr) is slightly double-peaked for *L* > *R*_||_ or even for the open-cylinder case (i) (i.e., *L* = ∞). A plausible reason for this is that the steric hindrance between the two arms of a ring polymer is minimized by (slight) helical organization. Second, *P*(Wr) develops a third peak at the origin (i.e., at Wr = 0) especially for ${\ell }_{{\rm{p}}}=40a\mathrm{,60}a$ and *L* = 20*a*, as labelled as (i’) in the middle panel; in this case, the three local minima at ${\rm{Wr}}\simeq \mathrm{0,}\,\pm 1$ are almost equally populated. The barrier height between left and right handedness is $\mathop{ < }\limits_{ \tilde {}}2{k}_{{\rm{B}}}T$. For the stiffest case, i.e., ${\ell }_{{\rm{p}}}\mathrm{=}\mathrm{100}a$, the local free energy minimum at Wr = 0 is 1–2*k*_B_*T* above the lower free energy at Wr = 0.

Similarly to the case of linear semiflexible chains in Fig. 3(A), ring semiflexible polymers tend to be helically organized if compressed moderately. Also, which local minimum is more likely populated depends on the value of ${\ell }_{{\rm{p}}}$. In contrast to the linear case, the barrier between the left and right handedness is not sensitive to ${\ell }_{{\rm{p}}}$ for the parameter ranges used in Fig. 3. This appears to be correlated with the emergence of a third peak at Wr = 0, which tends to lower the barrier, more so for a larger value of ${\ell }_{{\rm{p}}}$. When compressed sufficiently, the ring polymer is also back-folded, as described by the ring illustration (iii) in the middle column, corresponding to the green curve for *D* = 6*a* in the upper graph in Fig. 3(B). In this case, the peak at Wr = 0 disappears, since the balance is swayed toward a back-folded conformation. Also the ring chain crosses over itself once, meaning that the ring has Wr ≈ ±1. The typical conformation, however, does not resemble a “perfect” helix or spiral. (As the parameters are varied, Wr will change accordingly.)

A lesson from the results in Fig. 3 is that DNA molecules by themselves will not easily assume helical organization beyond their intrinsic helical structure. The persistence length of these molecules is ${\ell }_{{\rm{p}}}\approx 50\,{\rm{nm}}$ or 25 in units of their cross-sectional diameter or width, which is about 2 nm. For this parameter choice, however, the chain tends to be back-folded (iii) rather than to be helically organized. Note here that nonzero Wr does not necessarily mean perfect helical or spiral organization. It could represent a back-folded conformation as in illustration (iii) in Fig. 3.

In the case of bacterial chromosomes^3–6^, a more relevant scenario is supercoiling-induced helical organization. Because of supercoiling, the bacterial chromosome resembles a bottle brush, consisting of a backbone and many side loops (supercoiled plectonemes) radiating from it, as shown in Fig. 1 ^3,13^. Indeed, an earlier study showed that a bottle brush can be helically organized in a cylindrical space^18^.

Here, we have employed a bottle-brush polymer to unravel further the helical organization of bacterial chromosomes. The backbone of a bottle-brush polymer is either linear or circular with a sidechain emerging from every backbone monomer. In our consideration, both the backbone and the side chains are intrinsically flexible. Each sidechain is formed by *N*_*s*_ monomers of the same kind/size as the backbone monomer. The resulting bottle-brush consists of (*N* × *N*_*s*_ + *N*) monomeric units in total with *N* monomers in the backbone and (*N* × *N*_*s*_) in the sidechains.

The degree of helical organization will depend on parameter choices. To explore a wide parameter space, we have used various combinations of parameter values, including those that are relevant for the helical organization of bacterial chromosomes^3–6^. We have chosen them as follows: the backbone length *N* = 100, 200; the side chain length *N*_*s*_ = 10, 26, 40; the cylinder diameter *D* = 15, 18, 20, 30*a*; the cylinder length *L* = 30, 35, 40, 45, 50*a*, and ∞ (open cylinder). With these choices, the volume fraction of the monomers $0.1\mathop{ < }\limits_{ \tilde {}}\varphi \mathop{ < }\limits_{ \tilde {}}0.18$. Also, we have estimated the persistence ${\ell }_{{\rm{p}}}$ of the linear bottle brush when it is in a free space, following ref.^33^.

The persistence length ${\ell }_{{\rm{p}}}$ of a bottle brush can be obtained from the projection of the end-to-end vector of the backbone chain onto the *k*-th unit or normalized bond vector that connects two consecutive monomers at *k* and *k* + 1. When viewed as a function of *k*, the maximum of this quantity can be taken as the persistence length^33^. As for the case of semiflexible polymers, the chain stiffness will be one of important factors for governing helical organization of a bottle-brush polymer. In this work, the persistence length of bottle brushes is controlled by *N*_*s*_: For *N*_*s*_ = 10, 26, 30, 40, ${\ell }_{{\rm{p}}}\simeq \mathrm{20.1,}\,\mathrm{33.8,}\,\mathrm{37.3,}\,42.4a$, respectively. The resulting ${\ell }_{{\rm{p}}}$ value is larger than the *D* value used; recall that the ratio ${\ell }_{{\rm{p}}}/D$ is a determining factor for the helical organization of a semiflexible polymer presented in Fig. 3. Our parameter choice represents a sizeable range of helical tendency: from no or weak helical tendency up to strong helical tendency.

**Figure 4:**
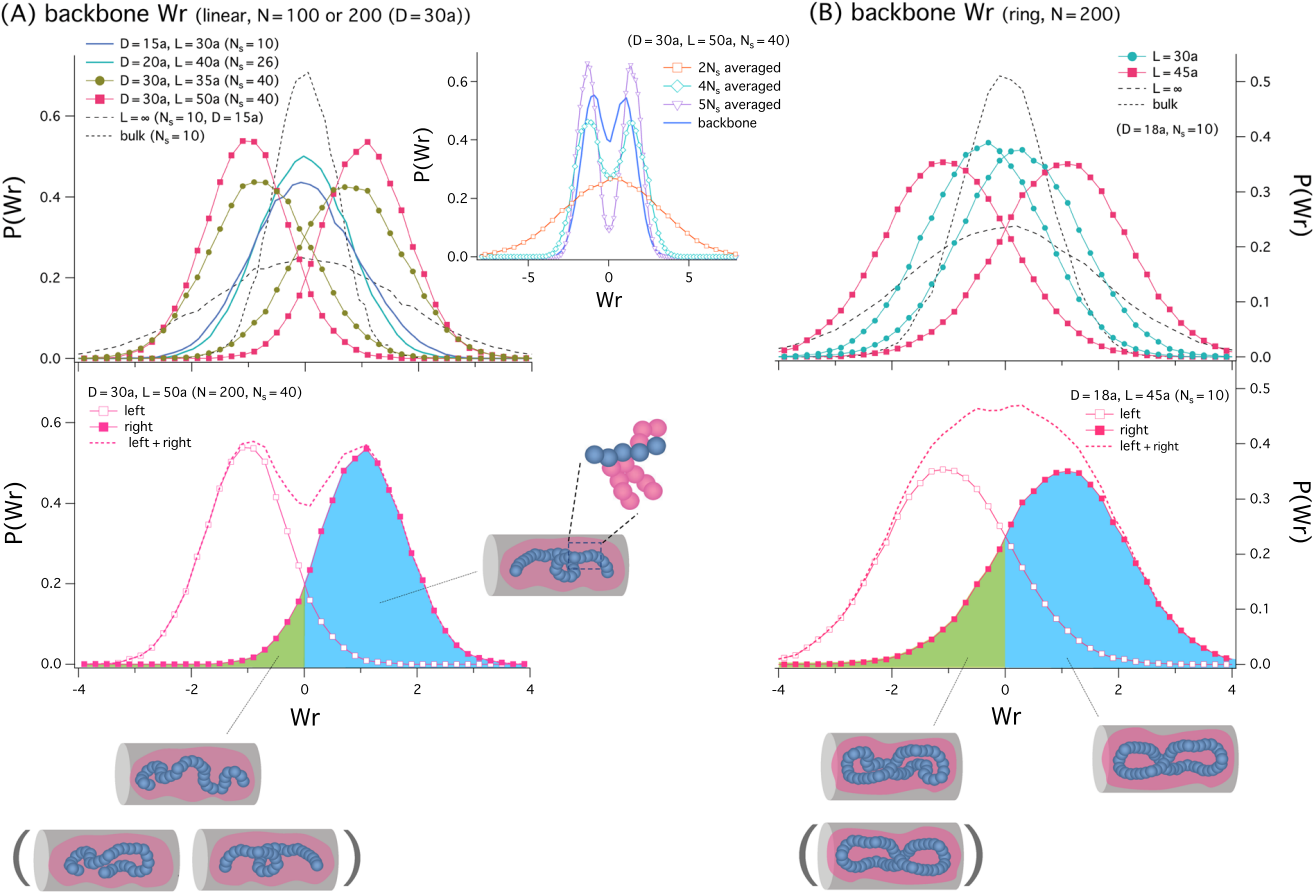
Probability distribution of Wr for a linear (**A**) and ring-shaped bottle brush (**B**), *P*(Wr). Here, we have chosen the parameters as: *N* = 100 (for *D* = 15*a*, 20*a*) and *N* = 200 (for *D* = 30*a*) for the linear-shaped bottle brush; *N* = 200 for the ring-shaped bottle brush. (**A**) The upper graph shows our results for *P*(Wr) for the backbone of a linear bottle brush for various combinations of *N*, *N*_*s*_, *D*, and *L* (see the legend). The linear bottle brush is back-folded, similarly to what the illustration (iii) in Fig. 3 suggests, and does not show preferred handedness in all cases, except for *N*_*s*_ = 40. For *N*_*s*_ = 40, left-handed and right-handed conformations are kinetically well separated even for the monomer volume fraction *ϕ* < 0.1 (for instances, *L* = 60, 74*a*). In this case, we obtained *P*(Wr) separately for the two initial conformations: left-handed and right-handed. The inset graph summarizes *P*(Wr) for the side chains of a linear bottle brush for *D* = 30*a*, *L* = 50*a*, and *N*_*s*_ = 40. We obtained *P*(Wr) by tracking the average positions of every successive 2*N*_*s*_, 4*N*_*s*_, and 5*N*_*s*_ monomers along side chains. As shown in the graph, the *P*(Wr) curve for the backbone (solid line without symbols) is positioned between those for side chains averaged over 4*N*_*s*_ monomers and 5*N*_*s*_ monomers. The behavior of *P*(Wr) for the backbone is consistent with that for the side chain except for the 2*N*_*s*_-averaged case. The bottom graph recaptures the curve for *N*_*s*_ = 40; also shown is the sum of left-handed and right-handed distributions, which is double peaked. Under different conditions, it is single peaked (see the bottom right for a ring analogue). The negative tail of an otherwise right-handed distribution (filled symbols) represents crumpled conformations as illustrated at the bottom; we have not observed a transition of an initially right-handed conformation into a left-handed one described inside the round brackets; in the brackets, a back-folded conformation that may occur under different conditions is also shown. (**B**) The upper graph here shows *P*(Wr) for the backbone of a ring bottle brush. We have chosen *D* = 18*a* and used a few choices of *L*: *L* = 30*a*, 45*a*, and ∞. Compared to the linear bottle-brush case in (A), the ring bottle brush shows a stronger tendency to be helically organized; a spiral pattern is observed for *N*_*s*_ = 10. When the cylinder becomes more spherical (i.e., for *L* = 30*a*), the helical tendency becomes weaker. The bottom graph shows the sum of left-handed and right-handed distributions for *L* = 45*a*; it is roughly single-peaked. Even in this case, the transition from an initially right-handed to left-handed conformation (inside the round brackets) was not observed in our simulations.

Figure 4 shows our results for the probability distribution of Wr, *P*(Wr), primarily for the backbone of a linear (A) and ring-bottle brush (B); see below for the side chains. In both cases (A) and (B), bottle brushes are helically organized only if they are confined in a closed cylindrical space. In free space or in an open cylinder (*L* = ∞), the graph of *P*(Wr) is single-peaked at Wr = 0, resulting in 〈Wr〉 = 0; recall 〈…〉 is an average with respect to the distribution *P*(Wr). The main difference between the free-space and open cylinder cases is that the width of *P*(Wr) is wider for the latter. This reflects the fact that the equilibrium size of the bottle brush is larger in the latter case.

The linear bottle brush does not show an obvious preferred spiral pattern except for *N*_*s*_ = 40 and *D*=30*a*, i.e., for the longest side chain chosen in Fig. 4; the backbone of the bottle brush is back-folded, similarly to what is shown in the illustration labelled as (iii) in Fig. 3, which diminishes the tendency for preferred handedness. For *N*_*s*_ = 40, however, we note that the left-handed and right-handed helical states are kinetically well separated. If initially in a left-handed helical state, the bottle brush tends to sample left-handed helical states over the simulation time scale until it equilibrates in the subspace spanned by left-handed conformations. The long side chains hinder the “global” motion of the backbone chain from a left-handed to right-handed state, for instance. By symmetry consideration, one can argue that left-handedness and right handedness are equally likely to occur. For the aforementioned kinetic reason, in this case, we obtained *P*(Wr) separately for the two initial conformations: left-handed and right-handed. Even for *ϕ* < 0.1 (for instances, *L* = 60*a*, 74*a*, corresponding to *ϕ* = 0.075, 0.061, respectively), the transition between opposite helical states was not observed in our simulations; when *ϕ* → 0 (i.e., *L* = ∞), however, the bottle is not helically organized.

Along this line, it is worth noting that the negative tail of *P*(Wr) for an otherwise positive-helically organized bottle brush in Fig. 4(A) (see the curve for *D* = 30*a* and *L* = 50*a*) results from chain crumpling as illustrated at the bottom of the lower graph in the figure. Under different conditions (e.g., *D* = 30*a*, *L* = 50*a*, *N*_*s*_ = 26), back-folding is responsible for this negative tail. A typical back-folded conformation is shown in the round brackets. The negative tail is not an indication of the transition between two opposite “perfect” helical states; a negative helical state is shown in the round brackets at the bottom of the lower graph in Fig. 4. (The value of Wr alone may not distinguish between back-folded and helical conformations.)

Even though sampling the entire right-handed and left-handed space is kinetically limited for a confined bottle-brush polymer, we can argue that it does not limit the applicability of our writhe results on physics grounds. First, by symmetry, it is obvious that the bottle brush does not have an intrinsic preference for one type of handedness over the other. It has a symmetric free energy landscape about Wr = 0, similarly to the semiflexible polymer case presented in the lower graph in Fig. 3. This allows us to sample either subspace spanned by one type of helical states. If the bottle brushes relaxes in the subspace, it is the shape of the barrier that is not well captured. For the helically-organized semiflexible chain in Fig. 3, labelled as (ii), the value of *P*(Wr) ≈ 0 for Wr ≈ 0, i.e, at or around the barrier location. In this case, the Wr distribution can be well approximated by a sum of left-handed and right-handed distributions sampled in respective spaces. The barrier is even higher for the bottle brush. This suggests that *P*(Wr) is even closer to zero for the bottle brush at Wr ≈ 0. One can thus obtain left-handed and right-handed distributions separately and add the two to get the full distribution. The higher the barrier is, the better the local equilibrium picture works. This justifies our local equilibrium approach to the Wr calculations of confined bottle-brush polymers.

The discussion above leads us to introduce a meaningful “population” average. Here we approximate it as a (normalized) sum of left-handed and right-handed distributions. It represents a population consisting of equal numbers of left-handed and right-handed bottle brushes.

The lower graph in Fig. 4(A) displays this sum, represented by a dashed line, along with the left-handed and right-handed curves, marked by open and filled squares, respectively. The sum has obvious double peaks. Under different conditions, the sum of opposite handed curves has a simple peak. See Fig. 4(B) and below for a representative single-peak sum.

The inset graph summarizes *P*(Wr) for the side chains of a linear bottle brush. We obtained *P*(Wr), tracking the average positions of every successive 2*N*_*s*_, 4*N*_*s*_, and 5*N*_*s*_ monomers along side chains. As shown in the graph, the *P*(Wr) curve for the backbone (solid line without symbols) is positioned between those for side chains averaged over 4*N*_*s*_ monomers and 5*N*_*s*_ monomers. The behavior of *P*(Wr) for the backbone is similar to that for the side chain for the parameters used except for the 2*N*_*s*_-averaged case. With different parameter choices, the degree of the similarity between the backbone and side-chain distributions can vary (data not shown).

The top graph in Fig. 4(B) shows *P*(Wr) for the backbone of a ring bottle brush. We have chosen *D* = 18*a* and used a few choices of *L*: *L* = 30*a*, 45*a*, and ∞. Compared to the linear bottle-brush case in Fig. 4(A), the ring bottle brush shows a stronger tendency to be helically organized; a spiral pattern is observed for *N*_*s*_ values as small as *N*_*s*_ = 10. When the cylinder becomes more spherical, the helical tendency is reduced in the sense that the two peaks are closer to the origin. This is well aligned with what is observed with a linear bottle brush in Fig. 4(A) and a semiflexible chain in Fig. 3.

For the parameter choice *L* = 45*a* (*D* = 18*a* and *N*_*s*_ = 10), the sum of left-handed and right-handed curves appears to be single-peaked as shown at the lower graph in Fig. 4(B). Even in this case, the transition between the two opposite helical states was not observed in our simulations. With different parameter choices, e.g., larger *N*_*s*_ values, however, the sum of left-handed and right-handed distributions becomes double-peaked; see Fig. 4 for a linear analogue. As in the corresponding linear case in Fig. 4(A), the transition between the two is a rare event. The negative tail in this case represents crumpled conformations as illustrated by a typical conformation at the bottom of Fig. 4(B). (What’s shown inside the round brackets is a typical conformation with an opposite helicity.) This is paralleled by the linear crumpled or back-folded conformation at the bottom of Fig. 4(A).

The results in Fig. 4(A,B) suggest that under cylindrical confinement the length of side chains is a determining factor for the helical organization of a bottle-brush polymer; they also highlight the significance of the ring topology of bottle brushes in enhancing their helical tendency, similarly to what was seen in semiflexible chains in Fig. 3. It is tempting to interpret this trend in terms of ${\ell }_{{\rm{p}}}$. Indeed the bottle brush has a stronger tendency to be helically organized for a larger value of *N*_*s*_, i.e., when ${\ell }_{{\rm{p}}}$ is larger. There is however a clear difference between semiflexible and bottle-brush polymers. While the kinetic barrier in the former is a few *k*_B_T, it is “insurmountably” high in the latter.

The helical organization of a bottle brush can be attributed to the way its side chains are arranged spatially. This interpretation is in part supported by the observation that a simple (linear or ring) flexible chain in a cylindrical space is not helically organized. To maximize the conformational entropy of the side chains, the entire bottle brush tends to be helically organized, either negatively or positively.

Our earlier discussion suggests that opposite helical states are kinetically well separated (see the bottom graph in Fig. 4 and the relevant discussion above). In this case, chain back-folding will not occur easily. All this means that the confined bottle brush tends to remain helically (and overall-linearly) organized. This observation is well aligned with the notion of ‘length-wise’ folding of bacterial chromosomes^8^. In a highly confined space, this linear-helical organization can be viewed as a regular folding pattern for the long bacterial chromosome.

For the combination of *L* = 45*a* and *D* = 18*a* used in Fig. 4, the aspect ratio of the cylinder is 45/18 = 2.5, similar to that for *E. coli* (see refs^9,10^ and relevant references therein). In this case, the average value of Wr and the deviation of Wr are estimated to be 〈Wr〉 = −1.03 and *σ*_Wr_ = 1.58 for the left-helical distribution; 〈Wr〉 = 1.02 and *σ*_Wr_ = 1.59 for the right-helical distribution; 〈Wr〉 = 0.01 and *σ*_Wr_ = 2.07, when the sum of the two is used. The value of 〈Wr〉 is sensitive to how it is calculated; it depends on whether it is averaged with respect to left-helical, right-helical distributions or the sum of the two with the last one possibly representing a population average. The existing data in the literature (e.g., those in ref. ^6^) are statistically not significant enough for a systematic comparison with simulation data, obtained by averaging over more than ten thousand ensembles. Furthermore, as detailed below, finite experimental resolution can generate undesired consequences on writhe measurements, thus limiting the correct interpretations of the measurements.

**Figure 5:**
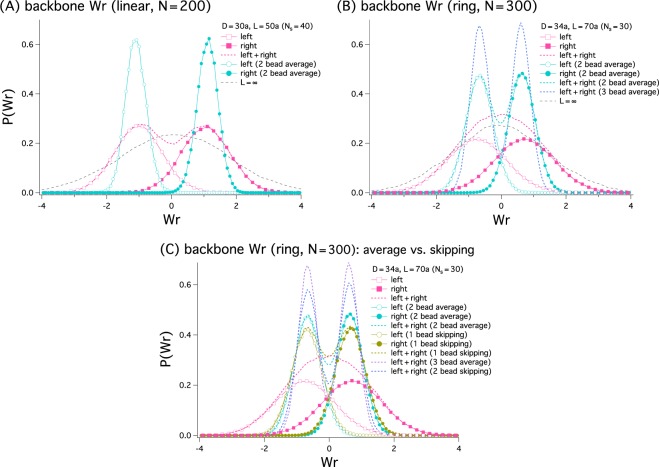
This figure shows how coarse-graining influences the Wr distribution, *P*(Wr), of linear (**A**) and ring bottle brushes (**B**). For the linear case, we have chosen *N* = 200, *N*_*s*_ = 40, *D* = 30*a*, and *L* = 50*a*; for the ring case, *N* = 300, *N*_*s*_ = 30, *D* = 34*a*, and *L* = 70*a*. (**A**) When all monomers are tracked, the left-handed (unfilled symbols) and right-handed (filled symbols) distributions overlap much. The sum of the two, represented by the dashed line, is double-peaked but is somewhat flat. The impact of coarse-graining is striking: When the mean position of the nearest two monomers is tracked, the left-handed and right-handed distributions become narrower; they barely overlap each other. In this case, the two peaks are kinetically better separated. (**B**) The unexpected effect of coarse-graining is well captured in this graph. The sum of the original left-handed and right-handed distribution is single-peaked. But the resulting curve after coarse-graining becomes double-peaked, more so for a larger degree of coarse-graining, i.e., when the position averaged over three consecutive beads is used. (**C**) This compares between the two coarse-graining methods for the ring case in (**B**) averaging over a few monomers and skipping a few monomers. Individual left-handed and right-handed distributions as well as their sum are shown. The distribution obtained by tracking the average position over two consecutive monomers compares favorably with the one obtained by tracking every other monomers. Similarly, averaging over three consecutive monomers is almost equivalent to skipping every successive two monomers. This trend persists in the linear case (data not shown).

The inset graph in Fig. 4 suggests that coarse-graining tends to make narrower the distribution of Wr. We have examined systematically how coarse-graining alters Wr distributions, *P*(Wr), and plotted our results in Fig. 5 for the backbone of linear (A) and ring bottle brushes (B). For this, we have chosen the parameters as: (A) *N* = 200, *N*_*s*_ = 40, *D* = 30*a*, and *L* = 50*a*; (B) *N* = 300, *N*_*s*_ = 30, *D* = 34*a*, and *L* = 70*a*. Here, coarse-graining means that the position of beads averaged over a few nearest beads is used in the calculation of Wr. As evidenced below, coarse-graining this way is qualitatively equivalent to skipping monomers. For instance, every other monomer can be tracked and taken into account. We note that coarse-graining gives better statistics.

When all monomers are tracked in the linear case in Fig. 5(A), the left-handed (curve with unfilled symbols) and right-handed (curve with filled symbols) distributions overlap much. The sum of the two, represented by the dashed line, is double-peaked but is somewhat flat. The impact of coarse-graining is “qualitative”: When the mean position of the nearest two monomers is tracked, the left-handed and right-handed distributions become narrower; they barely overlap each other. In this case, the two peaks become kinetically better separated upon coarse-graining.

The intriguing effect of coarse-graining is better illustrated in the graph in Fig. 5(B). The sum of the original left-handed and right-handed distribution is single-peaked. But the resulting curve after coarse-graining becomes double-peaked, more so for a larger degree of coarse-graining, i.e., when the position averaged over three consecutive beads is used; the Wr distribution becomes qualitatively different.

In a typical experimental setting, several loci along chromosomes are labelled with fluorescent molecules^4–6^. In a polymer model, this is equivalent to skipping monomers and track the rest in the computation of writhe. In Fig. 5(C), we have compared between the two coarse-graining methods for the ring case in (B): averaging over a few monomers and skipping a few monomers. For this, we have chosen the same parameters used in Fig. 5(B). The graph in Fig. 5(C) displays individual left-handed and right-handed distributions as well as their sum. The distribution obtained by tracking the average position over two consecutive monomers compares favorably with the one obtained by tracking every other monomers. Similarly averaging over three consecutive monomers is almost equivalent to tracking every third monomers. The trend is essentially the same in the linear case (data not shown).

A lesson from this observation is that chromosome experiments have to be interpreted with caution. Whether chromosome data will produce a single-peak or double-peak Wr distribution depends on the degree of coarse-graining set by experimental resolution. Also the width of distributions (i.e., left-handed or right-handed, or their sum) becomes narrower if more coarse-grained. Furthermore, a population average can wash out the kinetic barrier between left and right handedness, since it represents the sum of left-handed and right-handed distribution. Recall that the sum is single peaked at Wr = 0 even though left-handed and right handed distributions are kinetically well separated (see Figs. 4(B) and 5(B)).

## Conclusions and Discussions

In conclusion, we have clarified the physical origin of biomolecular helical organization by employing two possibly-complementary models: DNA-like semiflexible and chromosome-like bottle-brush polymers. Our results suggest that helical organization is not easily realized for DNA molecules beyond their intrinsic propensity to be double-helical in a biologically relevant parameter space. In contrast, bottle-brush polymers, resembling supercoiled bacterial chromosomes, tend to be helically organized in a cell-like closed cylindrical space, driven by the entropic ordering of their side chains in a spiral pattern, induced by cylindrical confinement.

Our analysis presented here indicates that one has to be cautious when interpreting chromosome data. If averaged over a population, the mean Wr tends to zero. This is, however, not the best representation of individual chromosomes, since they can get trapped in a certain helicity state for a “macroscopic” time scale, i.e., a time scale required for the entire rearrangement of the side chains. This implies that it is meaningful to consider the deviation of Wr for each helicity state, which is the “width” of the either or left peak of *P*(Wr) in addition to the sum of the two, which represents a population average.

Furthermore, our results also show how experimental resolution can limit the correct interpretation of chromosome data, since it influences the spread of writhe distributions and the kinetic separability of left-handed and right-handed peaks. With the aid of computer modelling based on a bottle-brush polymer, more meaningful writhe can be extracted from the data by appropriately choosing the simulation parameters and controlling the degree of course graining in concert with the experimental set-up.

## References

[1] Dill KA (1995). Principles of protein folding–a perspective from simple exact models. Protein Sci..

[2] Buehler MJ (2006). Nature designs tough collagen: Explaining the nanostructure of collagen fibrils. Proc. Natl, Acad. Sci.

[3] Le TBK, Imakaev MV, Mirny LA, Laub MT (2013). High-resolution mapping of the spatial organization of a bacterial chromosome. Science.

[4] Fisher JK (2013). Four-dimensional imaging of *E. coli* nucleoid organization and dynamics in living cells. Cell.

[5] Berlatzky IA, Rouvinski A, Ben-Yehuda S (2008). Spatial organization of a replicating bacterial chromosome. Proc. Natl. Acad. Sci.

[6] Yazdi NH, Guet CC, Johnson RC, Marko JF (2012). Variation of the folding and dynamics of the *Escherichia col*i chromosome with growth conditions. Mol. Microbiol..

[7] Chan H-K (2011). Densest columnar structures of hard spheres from sequential deposition. Phys. Rev. E.

[8] Wang X, Llopis PM, Rudner DZ (2013). Organization and segregation of bacterial chromosomes. Nat. Rev. Genet..

[9] Ha B-Y, Jung Y (2015). Polymers under confinement: single polymers, how they interact, and as model chromosomes. Soft Matter.

[10] Jun S, Wright A (2010). Entropy as the driver of chromosome segregation. Nat. Rev. Microbiol..

[11] Pelletier J (2012). Physical manipulation of the Escherichia coli chromosome reveals its soft nature. Proc. Nat. Acad. Sci. USA.

[12] Benza VG (2012). Physical descriptions of the bacterial nucleoid at large scales, and their biological implications. Rep. Prog. Phys..

[13] Hsu H-P, Paul W, Rathgeber S, Binder K (2010). Characteristic length scales and radial monomer density profiles of molecular bottle-brushes: simulation and experiment. Macromolecules.

[14] Paturej J, Sheiko SS, Panyukov S, Rubinstein M (2016). Molecular structure of bottlebrush polymers in melts. Sci. Adv..

[15] Calladine, C. R., Drew, H., Luisi, B. & Travers, A. *Understanding DNA: The Molecule and How it Works*, 3rd edit. (Academic Press, 2004).

[16] Jian H, Schlick T, Vologodskii A (1998). Internal motion of supercoiled DNA: Brownian dynamics simulations of site juxtapostion. J. Mol. Biol..

[17] Hayase Y, Sakaue T, Nakanishi H (2017). Compressive response and helix formation of a semiflexible polymer confined in a nanochannel. Phys. Rev. E.

[18] Chaudhuri D, Mulder BM (2012). Spontaneous helicity of a polymer with side loops confined to a cylinder. Phys. Rew. Lett..

[19] Wu F (2018). Cell boundary confinement sets the size and position of the *E. coli* chromosome. bioRxiv.

[20] Kremer K, Grest GS (1990). Dynamics of entangled linear polymer melts: A molecular-dynamics simulation. J. Chem. Phys..

[21] Grest GS, Kremer K (1986). Molecular dynamics simulation for polymers in the presence of a heat bath. Phys. Rev. A.

[22] Weeks JD, Chandler D, Andersen HC (1971). Role of repulsive forces in determining the equilibrium structure of simple liquids. J. Chem. Phys..

[23] de Gennes, P.-G. *Scaling Concepts in Polymer Physics* (Cornell University Press, 1979).

[24] Allison SA (1986). Brownian dynamics simulation of Wormlike chains. Fluorescence depolarization and depolarized light scattering. Macromolecules.

[25] Plimpton S (1995). Fast parallel algorithms for short-range molecular dynamics. J. Comput. Phys..

[26] Bates, A. D. & Maxwell, A. *DNA Topology* (Oxford University Press, 2005).

[27] Klenin K, Langowski J (2000). Computation of writhe in modeling of supercoiled DNA. Biopolymers.

[28] Reisner W, Pedersen JN, Austin RH (2012). DNA confinement in nanochannels: physics and biological applications. Rep. Prog. Phys..

[29] Jun S, Thirumalai D, Ha B-Y (2008). Compression and stretching of a self-avoiding chain in cylindrical nanopores. Phys. Rev. Lett..

[30] Wang Y, Tree DR, Dorfman KD (2011). Simulation of DNA extension in nanochannels. Macromolecules.

[31] Khorshid A, Amin S, Zhang Z, Sakaue T, Reisner W (2016). Nonequilibrium dynamics of nanochannel confined DNA. Macromolecules.

[32] Jung Y (2012). Ring polymers as model bacterial chromosomes: confinement, chain topology, single chain statistics, and how they interact. Soft Matter.

[33] Hsu H-P, Binder K, Paul W (2009). How to define variation of physical properties normal to an undulating one-dimensional object. Phys. Rev. Lett..

[34] Bakshi S, Choi H, Weisshaar JC (2015). The spatial biology of transcription and translation in rapidly growing *Escherichia coli*. Front. Microbiol..

[35] Jeon C, Hyeon C, Jung Y, Ha B-Y (2016). How are molecular crowding and the spatial organization of a biopolymer interrelated. Soft Matter.

